# Peroxiredoxin1, a novel regulator of pronephros development, influences retinoic acid and Wnt signaling by controlling ROS levels

**DOI:** 10.1038/s41598-017-09262-6

**Published:** 2017-08-21

**Authors:** Soomin Chae, Hyun-Kyung Lee, Yoo-Kyung Kim, Hyo Jung Sim, Yoorim Ji, Chowon Kim, Tayaba Ismail, Jeen-Woo Park, Oh-Shin Kwon, Beom-Sik Kang, Dong-Seok Lee, Jong-Sup Bae, Sang-Hyun Kim, Kyoung-Jin Min, Taeg Kyu Kwon, Mae-Ja Park, Jin-Kwan Han, Taejoon Kwon, Tae-Joo Park, Hyun-Shik Lee

**Affiliations:** 1 KNU-Center for Nonlinear Dynamics, School of Life Sciences, BK21 Plus KNU Creative BioResearch Group, College of Natural Sciences, Kyungpook National University, Daegu, 41566 South Korea; 2 School of Life Sciences, Ulsan National Institute of Science and Technology (UNIST), Ulsan, 44919 South Korea; 3 College of Pharmacy, Research Institute of Pharmaceutical Sciences, Kyungpook National University, Daegu, 41566 South Korea; 4 Department of Pharmacology, College of Medicine, Kyungpook National University, Daegu, 41944 South Korea; 5 Department of Immunology, School of Medicine, Keimyung University, Daegu, 42601 South Korea; 6 Department of Anatomy, College of Medicine, Kyungpook National University, Daegu, 41944 South Korea; 7 Department of Life Sciences, Pohang University of Science and Technology, Pohang, Kyungbuk 37673 South Korea

## Abstract

Peroxiredoxin1 (Prdx1) is an antioxidant enzyme belonging to the peroxiredoxin family of proteins. Prdx1 catalyzes the reduction of H_2_O_2_ and alkyl hydroperoxide and plays an important role in different biological processes. Prdx1 also participates in various age-related diseases and cancers. In this study, we investigated the role of Prdx1 in pronephros development during embryogenesis. Prdx1 knockdown markedly inhibited proximal tubule formation in the pronephros and significantly increased the cellular levels of reactive oxygen species (ROS), which impaired primary cilia formation. Additionally, treatment with ROS (H_2_O_2_) severely disrupted proximal tubule formation, whereas Prdx1 overexpression reversed the ROS-mediated inhibition in proximal tubule formation. Epistatic analysis revealed that Prdx1 has a crucial role in retinoic acid and Wnt signaling pathways during pronephrogenesis. In conclusion, Prdx1 facilitates proximal tubule formation during pronephrogenesis by regulating ROS levels.

## Introduction

The kidney is an indispensable homeostatic organ that maintains fluid and salt balance in the body^1^. The kidney functions by filtering and excreting wastes from the body^1, 2^. Three main structures are formed during kidney development, namely, the pronephros, mesonephros, and metanephros^3^, all of which originate from the intermediate mesoderm during embryogenesis^4^. The pronephros, mesonephros, and metanephros are structurally and functionally distinct, except that the nephron is common to all three structures^5^. The nephron comprises three basic components, namely, the glomerulus, tubule, and duct, and the role of each component in excreting wastes is different. The metanephros serves as the adult kidney in mammals, whereas the mesonephros acts as the adult kidney in amphibians. On the other hand, the pronephros is critical for embryonic development^6^. Although the adult kidney is distinct in different vertebrates, the underlying mechanism of kidney development is similar in zebrafish, frogs, mice, and humans^7^.

Kidney development is a complex process involving a series of steps that initiate from the intermediate mesoderm at the neurula stage of embryogenesis^3, 5^. Several signaling cascades, including bone morphogenetic protein, fibroblast growth factor, notch, Wnt, and retinoic acid (RA) pathways^4^, are involved in pronephros development. Wnts comprise a family of signaling proteins that bind to Frizzled (Fzd) receptors. Upon activation, Fzd receptors transduce signals to proteins belonging to the Dishevelled (Dsh) family^8^. Dsh proteins are phosphoproteins that consist of three functional domains, namely, a DIX domain at the N-terminus, a PDZ domain, and a DEP domain at the C-terminus^9^. Different combinations and interactions among these three domains determine the mode of Wnt signaling, which includes canonical versus non-canonical Wnt signaling^10^. Evidence indicates that canonical Wnt signaling plays an important role in kidney development, and inhibition of Wnt signaling impedes pronephros development in amphibians^8^. By contrast, Wnt/β-catenin-independent (non-canonical Wnt) signaling is responsible for proximal tubule formation during pronephrogenesis^8, 9, 11^. RA signaling is also crucial for pronephros development, and its inhibition results in pronephros abnormalities^12–14^. Pronephros development is associated with the expression of number of different specific genes and their corresponding transcribed and translated products. Any misregulation in the expression of these genes or abnormalities in the corresponding transcription and translation can lead to developmental defects in the pronephros^15^.

The peroxiredoxin (Prdx) family comprises thiol-based proteins that function as antioxidants^16^. Prdx proteins catalyze the reduction of different peroxide substrates, and are crucial for H_2_O_2_-mediated cell signaling^17^. Prdx has six isoforms that are categorized into three subclasses based on the number and position of the cysteine residues, namely, the 2-Cys, atypical 2-Cys, and 1-Cys subclasses. Prdx1 through 4 belong to the 2-Cys subclass; Prdx5 belongs to the atypical 2-Cys subclass; and Prdx6 belongs to the 1-Cys subclass^17, 18^. Prdx1 through 6 possess conserved cysteine residues and undergo oxidation–reduction cycles^18^. In addition to their functions as antioxidants, Prdx proteins are also involved in various physiological processes^19, 20^.

Peroxiredoxin1 (Prdx1), a typical 2-Cys Prdx that contains two conserved cysteines, catalyzes the reduction of H_2_O_2_ and alkyl hydroperoxide^21^. During normal Prdx1 antioxidant function, the conserved N-terminal cysteine (Cys-52) residue is selectively oxidized to Cys-SOH by H_2_O_2_. Oxidized Prdx then forms an intermolecular disulfide bond with the conserved thiol group (-SH) at the C-terminus (Cys-178) of the other subunit in the head-to-tail homodimer^22^. During this process, thioredoxin (Trx) reduces the disulfide bond^23^. Prdx1 also plays a critical role in the progression of different types of cancer, and several studies have proposed therapies aimed at halting or slowing down cancer growth^16, 22^. In addition to its protective effects against reactive oxygen species (ROS) or oxidative stress, Prdx1 also influences downstream signaling pathways during organogenesis^24^.

In the present study, we demonstrate that Prdx1, an antioxidant enzyme, plays a crucial role in pronephros development. Loss of Prdx1 resulted in abnormal proximal tubule formation in the pronephros. Catalytic mutants of Prdx1 (C53S, C173S, and C53S/C173S) were unable to dimerize, thus failing to rescue the Prdx1 knockdown-induced disruption of proximal tubule formation. In addition, treatment with ROS severely disrupted proximal tubule formation, which was restored by Prdx1 overexpression. Moreover, Prdx1 modulated RA and Wnt signaling during pronephros development. In conclusion, Prdx1 is essential for proximal tubule formation during pronephrogenesis.

## Results

### Prdx1 knockdown arrests proximal tubule formation in the pronephros during embryogenesis

To analyze the Prdx1 expression pattern, RT-PCR and whole-mount *in situ* hybridization were performed on *X. laevis* embryos. The Prdx1 transcript was maternally expressed (stage 0), and its expression gradually increased until the tadpole stage of development (Fig. S1A). Whole-mount *in situ* hybridization analysis at different developmental stages (NF stage 8, 14, 16, 22 and 33) showed that *prdx1* was highly and predominantly expressed in the forebrain, eye, multiciliated cells, and pronephros of developing embryos (Fig. S1B). *prdx1* was also expressed in the intermediate mesoderm, which comprises the proximal compartments that develop into the proximal tubules (Fig. S1B).

To investigate the role of Prdx1 in pronephros development, we performed knockdown studies using *prdx1* antisense morpholino oligonucleotides (MOs) and to verify the specificity of *prdx1* MO, embryos at two-cell stage were injected with *prdx1* MO and prdx1* RNA that failed to bind to the *prdx1* MOs. The translation product for WT *prdx1* RNA was significantly reduced by *prdx1* MO but no marked reduction was observed for prdx1* RNA translated product (Fig. S2E). Hence, it is confirmed that *prdx1* is the only affected transcript by *prdx1* MO. *prdx1* MOs were injected into both blastomeres of two-cell stage embryos, and the phenotypes of the morphant embryos were analyzed. Embryonic development of *prdx1* morphants was abnormal, as evident by shorter trunks and smaller heads compared with the control MO-injected embryos (Fig. S2A). Since, MO injections at two-cell stage cause significant defects in axis formation and development of the notochord, somites and dorsal aorta in addition to the kidney, so to avoid the ambiguity that kidney defects arise directly from *prdx1* knockdown rather than secondarily caused by defects in other structures, we injected *prdx1* MO into a V.2.2 blastomere at 16-cell stage that is a major contributor for pronephros (Fig. S2C). Interestingly embryos exhibited similar developmental abnormalities as were observed for *prdx1* MO injections at two-cell stage (Fig. S2C). These results confirmed that the Prdx1 is essential for pronephros development and knockdown of Prdx1 led to abnormalities in embryonic kidney development.

The role of Prdx1 in pronephros development was further examined using hematoxylin and eosin-stained transverse sections of control and *prdx1* morphant embryos. *prdx1* MO-injected embryos displayed malformed or undifferentiated internal organs that appeared to be scattered compared with control MO-injected embryos (Fig. S2D). The analysis of transverse sections showed that the pronephros was poorly developed in *prdx1* MO-injected embryos compared with control MO-injected embryos.

**Figure 1:**
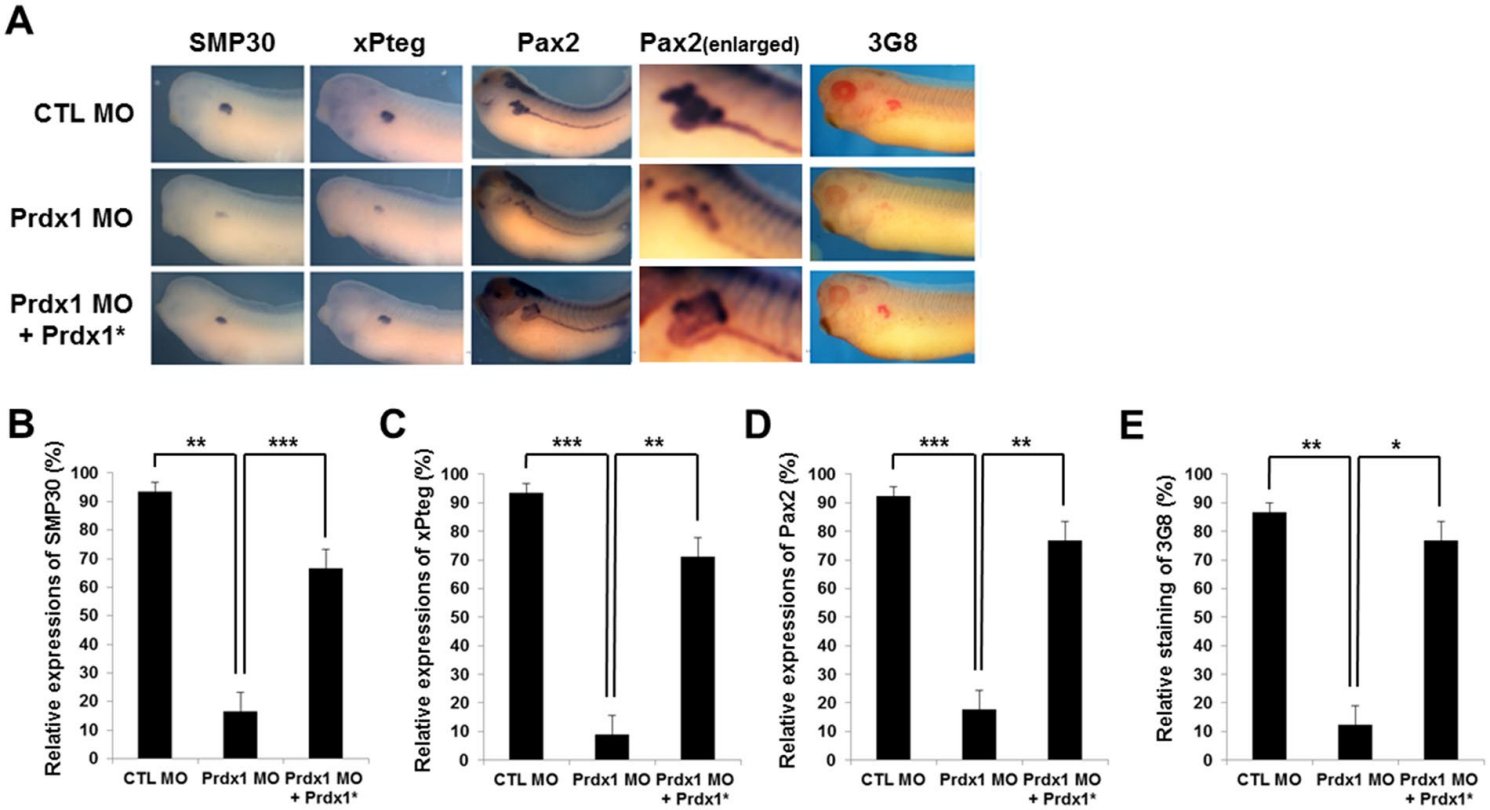
Inhibition of proximal tubule formation in the pronephros by the loss of Prdx1 and its rescue by Prdx1* RNA co-injection. (**A**) *prdx1* MOs (40 ng) were injected into both blastomeres of two-cell stage embryos. Embryos at the stage 33 were used for whole-mount *in situ* hybridization and immunohistochemistry. Proximal tubules in the pronephros were visualized by whole-mount *in situ* hybridization using *smp30*, *xPteg*, and *pax2* probes and by immunohistochemistry using a 3G8 antibody. Expression of the proximal tubule-specific markers *smp30* (**B**), *xPteg* (**C**), and *pax2* (**D**) in *prdx1* MO-injected embryos compared with the controls. Co-injection with prdx1* RNA rescued the decreased expression. The immunohistochemistry for 3G8 patterns were similar with whole-mount *in situ* hybridization (**E**). *prdx1* morphants exihibited inhibition of proximal tubule formation only while intermediate, distal and connecting tubules were not affected by loss of *prdx1*.

Next, we analyzed the Prdx1 morphant embryos using the proximal tubule-specific markers *smp30* ^25^, *xPteg* ^26^, and *pax2* ^27^ for whole-mount *in situ* hybridization. The levels of *smp30, xPteg*, and *pax2* were significantly lower in *prdx1* MO-injected embryos at the stage 33 than in control MO-injected embryos (Fig. 1A–E). As shown in Fig. 1A, the morphant phenotypes were rescued by co-injection with prdx1* RNA that failed to bind to the *prdx1* MOs. Interestingly, the inhibitory effects of the *prdx1* MOs were observed only in the proximal tubules (Fig. 1A–E). *pax2* was widely expressed in the distal, connecting, and proximal tubules in the developing pronephros. However, decreased *pax2* expression in *prdx1*-knockdown embryos was observed only in the proximal tubules, and not in intermediate, distal and connecting tubules (Fig. 1A,D). Similar results were obtained by immunohistochemistry using a 3G8 antibody that specifically stained the proximal tubules (Fig. 1A,E), while 4A6 antibody staining of intermediate, distal and connecting tubules of prdx1 morphants were not affected at all (Fig. S2F). The proximal tubule defects were rescued by co-injection with prdx1* RNA (Fig. 1A–E), supporting previous results that showed proximal tubule defects are due to the loss-of-function phenotype of Prdx1.

### Conserved cysteine residues in Prdx1 that are responsible for its function as an antioxidant are essential for pronephrogenesis

**Figure 2:**
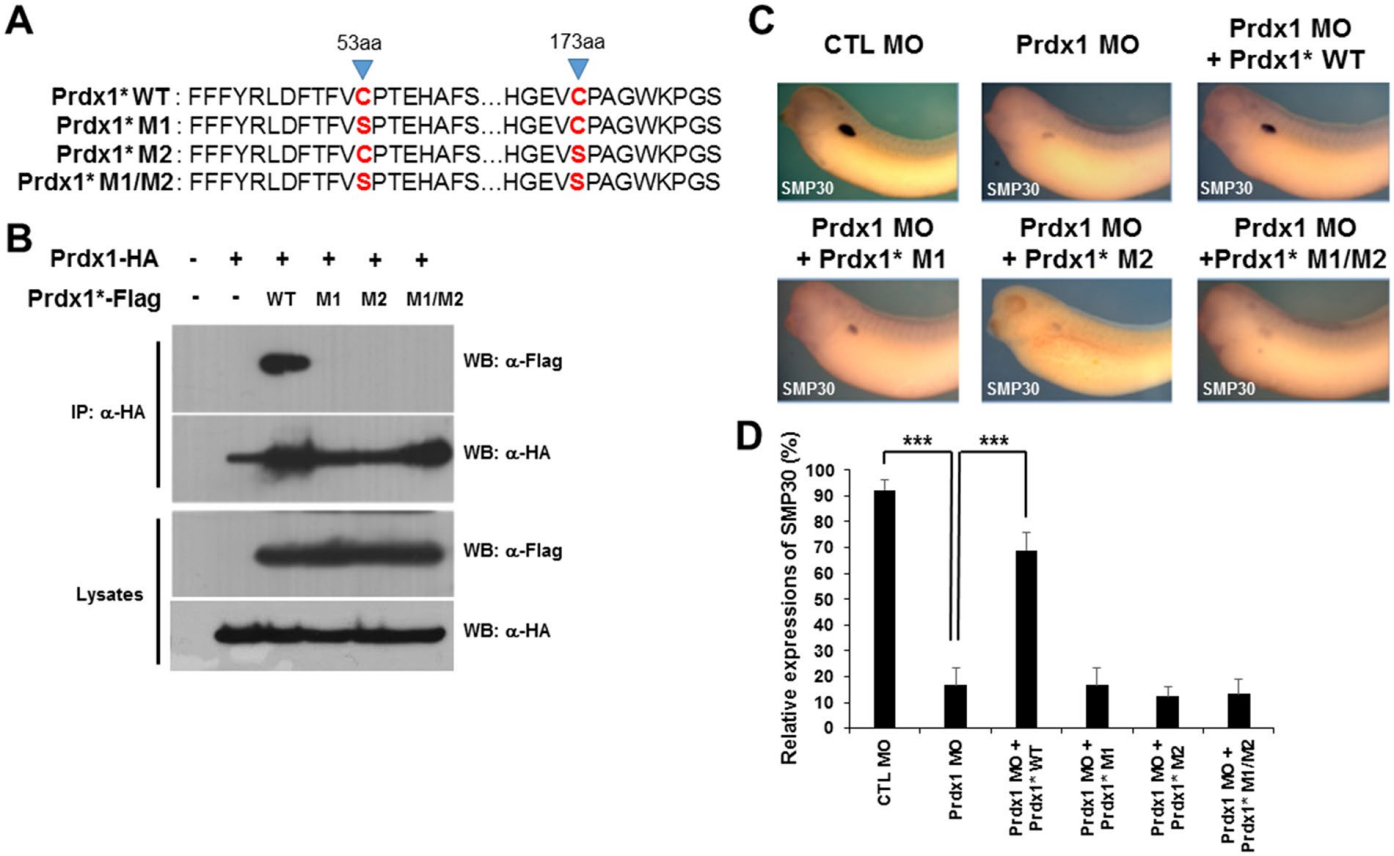
Conserved cysteines in Prdx1 are essential for pronephros development. (**A**) Three *prdx1* mutants, namely, C53S (M1), C173S (M2), and C53S/C173S (M1/2), were subcloned using PCR-based site-directed mutagenesis. (**B**) Flag-tagged Prdx1* or HA-tagged *prdx1* (200 pg) was co-injected into both blastomeres of two-cell stage embryos. Lysates from stage 12 embryos were used for immunoprecipitation. WT Prdx1 only showed in immune complex whereas mutant Prdx1 did not. (**C**) *prdx1* MOs (40 ng) were co-injected with WT or mutant *prdx1* (M1, M2 or M1/M2) into both blastomeres of two-cell stage embryos. Embryos at the stage 33 were used for whole-mount *in situ* hybridization, and proximal tubules in the pronephros were visualized with a *smp30* probe. Inhibited proximal tubule formation in prdx1 morphants was rescued by WT *prdx1* but not with mutant prdx1* RNAs. (**D**) Graphical demonstration of *smp30* expression in embryos co-injected with WT or mutant *prdx1* (M1, M2 or M1/M2) and *prdx1* MOs compared with the controls.

Prdx1, which is well known for its antioxidant properties in vertebrates, undergoes oxidative and reductive reactions within cells^28^. The function of Prdx1 as an antioxidant depends on its two conserved cysteine residues, namely, Cys-53 and Cys-173. Alterations in these conserved cysteine residues result in abnormalities. To determine the roles of these conserved cysteine residues in pronephros development during embryogenesis, we generated three Prdx1 mutants by changing the peroxidatic and resolving cysteine residues (Cys-53 and Cys-173, respectively) to serine, which disrupted the antioxidant function of Prdx1 (Fig. 2A).

To determine whether these Prdx1 mutants can bind to each other in embryos, we co-injected a Prdx1*-Flag-tagged mutant (WT, M1, M2, or M1/M2) with WT Prdx1-HA mRNA and performed immunoprecipitation using an anti-HA antibody. Flag-tagged WT Prdx1 (Prdx1*-Flag WT) associated with HA immunocomplexes (Figs 2B and S4). However, immunoreactive signals corresponding to the Flag-tagged Prdx1* mutants (M1, M2, and M1/M2) were not detected (Fig. 2B), indicating that the conserved cysteine residues in Prdx1 have roles similar to those in *X. laevis* embryos.

The roles of the conserved cysteine residues in pronephrogenesis were further analyzed by microinjecting the *prdx1* MOs with mutant prdx1* RNA (WT, M1, M2, or M1/M2). Embryos were analyzed by whole-mount *in situ* hybridization using a probe against the proximal tubule marker *smp30*. The *prdx1* MO-mediated inhibition of proximal tubule formation was rescued by injection with WT *prdx1*, but not with mutant prdx1* RNAs (Fig. 2C,D). These results clearly demonstrate that the peroxidase function of Prdx1 is critical for pronephros development.

### Prdx1 functions downstream of RA signaling during pronephrogenesis

**Figure 3:**
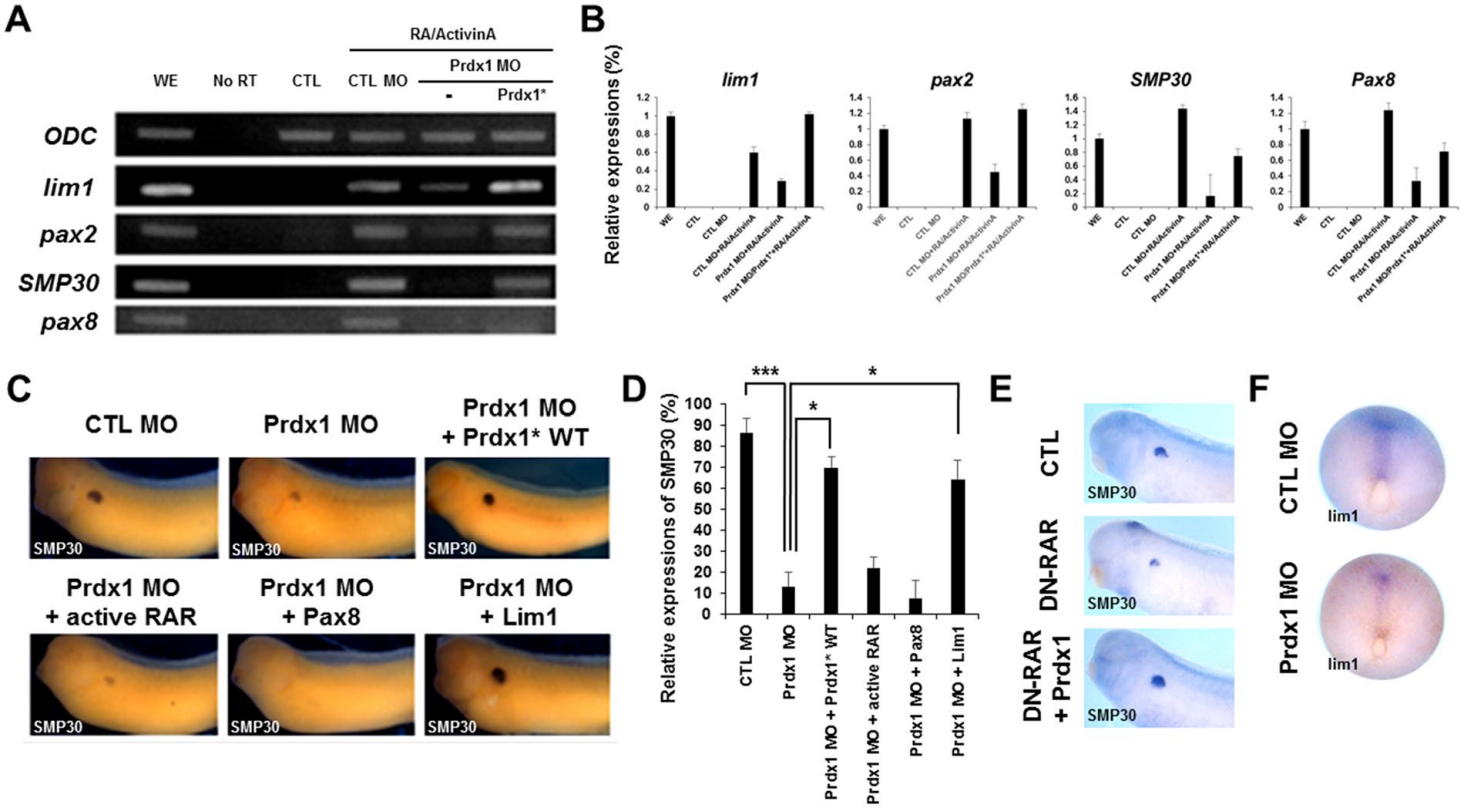
Prdx1 functions downstream of the RA signaling pathway during proximal tubule formation in the pronephros. (**A**) *prdx1* MOs (40 ng) were injected into both blastomeres of two-cell stage embryos. Animal caps were removed from the injected embryos at the stage 8.5 and incubated with 10 ng/mL activin A, followed by 10^−4^ M all-trans RA for 3 h. Animal caps were collected from embryos at the stage 33 and used for RT-PCR. RT-PCR results showed the significant reduced expression of *lim1*, *pax2*, *smp30*, and *pax8* in prdx1 MO-injected as compared with RA/activin induced. The decreased expression of pronephros markers was rescued by prdx1* RNA. Ornithine decarboxylase (*odc*) was used as the loading control. A no-RT template in the absence of reverse transcriptase was used as the control. WE, whole embryo; CTL, control animal caps; CTL MO, control MO-injected animal caps. (**B**) RT-PCR examination of *lim1, pax2, smp30* and *pax8* expression was confirmed by real time PCR. Significant lower expression level was observed for pronephros markers in the *prdx1* MO-injected embryos that was rescued by co-injection with prdx1* RNA except for *pax8*. WE, whole embryo; CTL, control animal caps; CTL MO, control MO-injected animal caps. (**C**) *prdx1* MOs (40 ng) were co-injected with wild-type *prdx1, RARα-vp16* (active *rar*), *pax8*, or *lim1* (400 pg) into both blastomeres of two-cell stage embryos. Embryos at the stage 33 were used for whole-mount *in situ* hybridization, and proximal tubules in the pronephros were visualized with a *smp30* probe. The pronephros abnormalities of *prdx1* morphants were rescued by *lim1* mRNA co-injection but not by the activation of active RA receptors or *pax8*. (**D**) Graphical representation of pronephros development in *prdx1* MO-injected embryos and embryos co-injected with *lim1*, active RA receptors and *pax8* compared to the control embryos (injected with control MO). Pronephros defects were significantly rescued by lim1 mRNA co-injection. (**E**) Embryos at two-cell stage were injected with dominant-negative retinoic acid receptor (*DN-RAR*) with or without *prdx1*. *DN-RAR*-inhibited pronephros development was rescued by *prdx1 overexpression*. (**F**) Expression of pronephros marker *lim1* was observed at stage 12.5 in embryos injected with CTL MO and *prdx1* MO. *lim1* expression was dramatically reduced in Prdx1 morphants.

RA signaling is essential for pronephros development^29^. The treatment of *X. laevis* gastrulae with trans-RA and activin can induce the differentiation of pluripotent ectodermal cells to the pronephros^29^. Moreover, pronephros development is impaired when RA signaling is inhibited^29^. To investigate the role of Prdx1 as a mediator of RA signaling during pronephros development, we performed an *in vitro* kidney induction assay. Animal cap cells (stem cell-like) were removed from the injected embryos at the stage 8.5 and sequentially treated with activin A (10 ng/mL) and all-trans RA (10^−4^ M) as previously described^29^. Animal cap cells were then induced to form the pronephric mesoderm, pronephric tubules, and glomus^29^. Next, we examined the levels of several pronephros markers during pronephros development by reverse transcription-PCR (RT-PCR) and real time PCR. The levels of *lim1, pax2*, *smp30*, and *pax8* were significantly lower in the *prdx1* MO-injected embryos than in the control MO-injected embryos (Fig. 3A,B). The decreased expression of *lim1, pax2,smp30* and *pax8* was rescued by co-injection with prdx1* RNA (Fig. 3A,B). These results indicate that Prdx1 functions as a one of significant modulator of RA signaling for pronephrogenesis.

RT-PCR and real time PCR results were validated by conducting epistatic experiments, which restored the decreased expression of the pronephric markers upon *prdx1* knockdown. *prdx1* MOs were co-injected with various RNAs [WT *prdx1*, *RARα-vp16* (constitutively active RA receptor), *pax8*, or *lim1*] into both blastomeres of two-cell stage embryos. Embryos at the stage 33 were analyzed by whole-mount *in situ* hybridization using a *smp30* probe. The defects in the pronephros of *prdx1* morphants were rescued by *lim1* mRNA co-injection, but not by the activation of the active RA receptor or *pax8* (Fig. 3C,D). To discern affected stages of kidney development by *prdx1* activity, we showed *lim1* expression at stage 12.5 embryos injected with control MO and *prdx1* MO (Fig. 3F). Interestingly, *lim1* expression was markedly reduced in *prdx1* morphants suggesting that early retinoic acid signaling was also affected (Fig. 3F). Thus, RT-PCR results and epistatic analysis showed that Prdx1 regulates pronephrogenesis by acting as a downstream mediator of RA signaling. To prove the epistatic analysis, we injected dominant-negative retinoic acid receptor (*DN-RAR*) into both blastomeres of two-cell staged embryos with or without *prdx1* RNA and it is clearly evident that DN-RAR-inhibited pronephros development was partially rescued by *prdx1* (Fig. 3E). Hence, it is confirmed that Prdx1 acts as a mediator of RA signaling during pronephros development.

### Prdx1 regulates pronephros development via the Wnt signaling pathway

**Figure 4:**
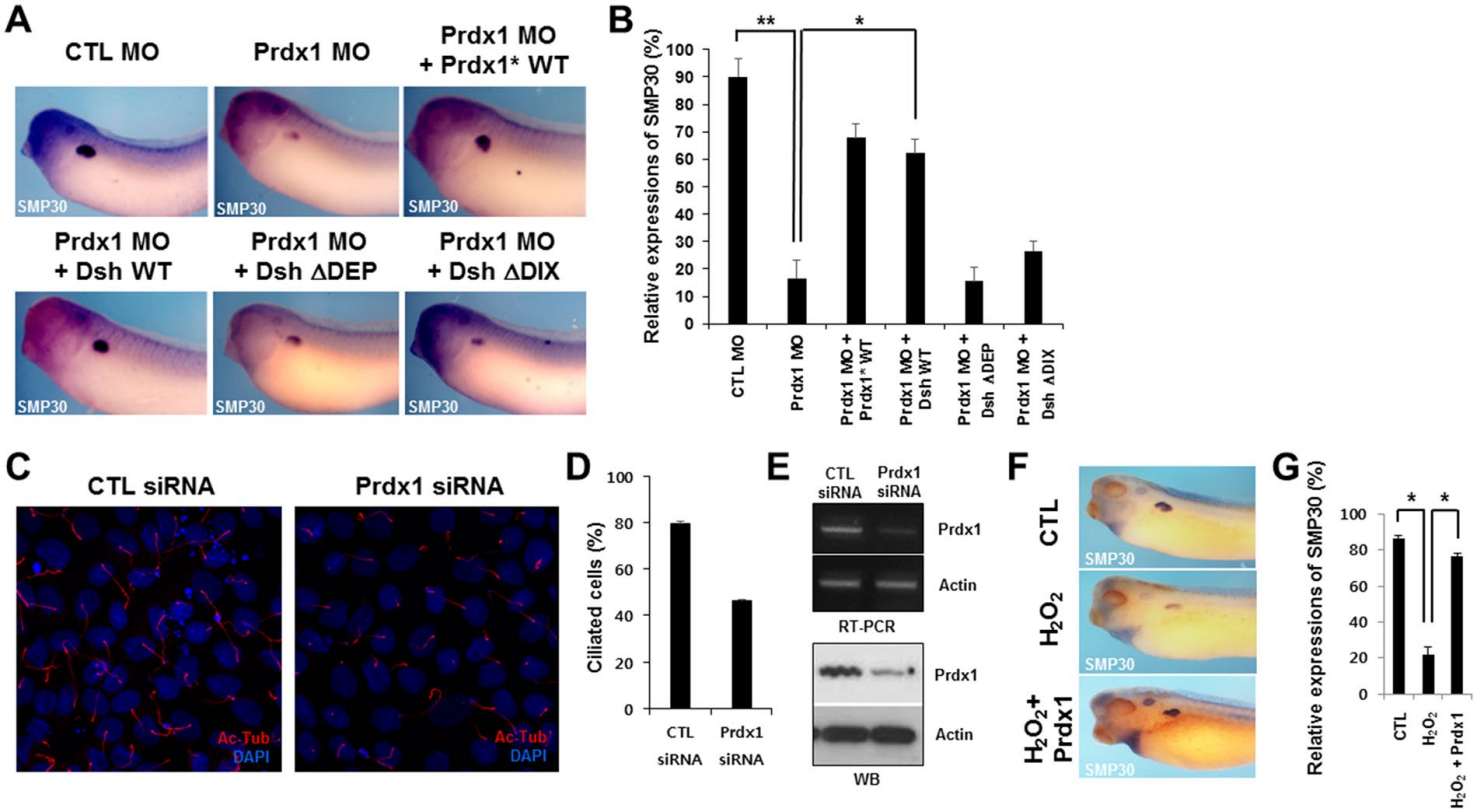
Prdx1 regulates pronephrogenesis via the Wnt signaling pathway by modulating ROS levels in *X. laevis* embryos. (**A**) *prdx1* MOs and WT prdx1*, WT *Dsh* (Dsh WT), *DshΔDEP*, or *DshΔDIX* were co-injected into both blastomeres of two-cell stage embryos. Embryos at the stage 33 were used for whole-mount *in situ* hybridization, and proximal tubules in the pronephros were visualized with a *smp30* probe. Pronephros development recovered only in embryos co-injected with WT prdx1* or Dsh WT. (**B**) Graphical demonstration of pronephros development in embryos co-injected with *prdx1* MOs and WT prdx1*, *Dsh* WT, *DshΔDEP*, or *DshΔDIX*. (**C**) MDCK cells were transfected with either 10 nM *prdx1* or control siRNAs. Primary cilia were visualized using an acetylated-tubulin (Ac-Tub) antibody. Cell nuclei were stained with DAPI. Numbers of cilia cells were markedly reduced in MDCK cells by *prdx1* siRNA mediated knockdown of *prdx1*. (**D**) Graphical representation of ciliated cells transfected with control siRNA and *prdx1* siRNA. *prdx1* knockdown significantly reduced the number of cilia in MDCK cells. (**E**) Specificity of *prdx1* knockdown in MDCK cells transfected with Prdx1siRNA was confirmed by RT-PCR examination as well as western blot studies. Significant reduction in levels of prdx1 mRNA and proteins was observed in MDCK cells transfected with Prdx1siRNA. (**F**) Control and *prdx1*-injected (200 pg) embryos were treated with 2 μM H_2_O_2_ at stage 8.5. Embryos at the stage 33 were used for whole-mount *in situ* hybridization, and proximal tubules in the pronephros were visualized with a *smp30* probe. H_2_O_2_-disrupted proximal tubule formation in the pronephros, and abnormal proximal tubule formation was rescued in Prdx1-injected embryos. (**G**) Graph showing the pronephros development in embryos co-treated with H_2_O_2_ and prdx1. Reduced expression of smp30 in H_2_O_2_ treated embryos was rescued by *prdx1*.

To investigate whether Prdx1 regulates pronephrogenesis via the Wnt signaling pathway, we generated two Dsh deletion constructs, designated Dsh*ΔDEP* and Dsh*ΔDIX*. The DIX domain of Dsh is a key regulator of canonical Wnt signaling, whereas the DEP domain activates non-canonical Wnt signaling^30, 31^. We co-injected the *prdx1* MOs and prdx1* RNA, with either *Dsh WT* or the Dsh deletion constructs (*DshΔDEP* or *DshΔDIX*), and then monitored pronephros development by whole-mount *in situ* hybridization using a *smp30* probe. The *prdx1* MO-induced inhibition of pronephros development was restored in embryos co-injected with *Dsh* WT RNA, but not with *DshΔDEP* or *DshΔDIX* RNA (Fig. 4A,B). These results indicate that Prdx1 regulates pronephrogenesis by influencing both canonical and non-canonical Wnt signaling.

### Loss of Prdx1 leads to the formation of fewer primary cilia and increased ROS levels in MDCK cells

Next, we sought to elucidate the molecular mechanism underlying the Prdx1 regulation of Wnt signaling during pronephros development. Both canonical and non-canonical Wnt signaling pathways are required for normal ciliary function^32–34^. In addition, abnormal ciliary function is the primary cause of kidney defects during development^35^. Therefore, we speculated that Prdx1 is necessary for cilia formation. To address this, we evaluated cilia formation in Madin-Darby, canine kidney (MDCK) cell lines after *prdx1* knockdown. We transfected MDCK cell lines with 10 nM control or *prdx1* siRNAs and observed the effects on primary cilia formation. As previously reported, the ROS levels were higher in *prdx1*-silenced MDCK cells than in the control, thus validating the efficacy of the siRNA-mediated knockdown of *prdx1* (Fig. S3). Cilia were then stained using an acetylated-tubulin antibody. The number of cilia in MDCK cells transfected with *prdx1* siRNAs was significantly lower than in cells transfected with control siRNAs (Fig. 4C–E). To validate that the Prdx1 overexpression inhibit the effects caused by increased ROS production, we treated MDCK cells with 100 μM H_2_O_2_ and observed the effect H_2_O_2_ on the expression of phosphorylated kinases (P-AKT, P-AMPKα and P-ERK). We found that treatment of MDCK cells with H_2_O_2_ enhanced the phosphorylation of AKT (P-AKT) while the phosphorylation of AMPKα (P-AMPKα) was reduced but the H_2_O_2_ treatment of MDCK cells transfected with *prdx1* exhibited reverse expression *i.e*. P-AKT was downregulated while P-AMPKα showed upregulated expression (Fig. S3B). Induced P-ERK by H_2_O_2_ was not affected by expression of Prdx1 in transfected MDCK cells. These altered level of expression by prdx1 transfection confirmed that prdx1-inhibited the H_2_O_2_ mediated responses (Fig. S3B). Taken together, these data indicate that an uncontrolled increase in the ROS levels results in ciliary defects that lead to severe malformations in the pronephros and Prdx1 significantly inhibits the responses induced by increased ROS production.

### Prdx1 overexpression rescues the ROS-induced disruption of proximal tubule formation in the pronephros

Kidney dysfunction associates with increased ROS levels. Prdx1 regulates the production of ROS and protects tissues from the damaging effects of ROS^36, 37^. We treated control or *prdx1*-overexpressed stage 8.5 embryos with H_2_O_2_ (an inducer of ROS production), evaluated the effects of ROS overproduction on proximal tubule formation, and examined the role of Prdx1 in ROS regulation and pronephros development. For this purpose we treated embryos with different concentration of H_2_O_2_ and found that embryos treated with 2 μΜ concentration of H_2_O_2_ did not exhibit defects in most organs including pronephros but severe malformations in majority of the organs including pronephros were observed for embryos treated with higher concentrations of H_2_O_2_. The treatment of embryos with H_2_O_2_ inhibited proximal tubule formation in the pronephros (Fig. 4F,G), indicating that ROS affects proximal tubule formation in the pronephros. In addition, the abnormal phenotype was restored by *prdx1* overexpression (Fig. 4F,G). Overall, these data indicate that Prdx1 regulates the production of ROS, which is important for normal proximal tubule formation in the pronephric duct. In conclusion, Prdx1 is essential for pronephros development during primary cilia formation, and it functions by regulating ROS levels.

## Discussion

Prdx1 is an antioxidant protein involved in many biological processes. Previous studies indicate that Prdx1 not only regulates the cellular ROS levels; it also functions in different cell signaling pathways and plays a role in vertebrate development^38^. In the current study, we demonstrated a role for Prdx1 in pronephros development during *X. laevis* embryogenesis and showed that Prdx1 is essential for *X. laevis* pronephrogenesis.

A number of genes expressed in the pronephros regulate its development^39^. Our results showed that *prdx1* is upregulated in the developing pronephros. The MO-mediated knockdown of *prdx1* resulted in the decreased expression of the proximal tubule-specific markers *smp30, xPteg*, and *pax2* (Fig. 1). The developmental defects in the pronephros due to *prdx1* depletion were further confirmed by immunohistochemistry using the proximal tubule-specific antibody 3G8 (Fig. 1). Moreover, rescue experiments highlighted the importance of *prdx1* in pronephros development. The reduced expression of proximal tubule-specific markers and the inhibition of pronephros formation were restored by co-injection with prdx1* RNA (Fig. 1). In summary, our findings clearly demonstrate that Prdx1 is essential for *X. laevis* pronephrogenesis.

The antioxidant properties of Prdx1 as a regulator of cellular ROS levels have been established^16^. Structural analysis of Prdx1 showed two conserved cysteine residues of Prdx1, namely, Cys-52 and Cys-178, to be largely responsible for the antioxidant properties of Prdx1^18^. In this study, the roles of these conserved cysteine residues in pronephros development were evaluated using Prdx1-Flag mutants carrying Cys-53- or Cys-173-to-serine point mutations. These mutants were co-injected with WT *prdx1* RNA. Results of the immunoprecipitation assay showed that only WT *prdx1*, and not the mutants, was present in the immunocomplexes (Fig. 2A,B). In addition, co-injection of embryos with the *prdx1* MOs and the prdx1* mutant RNAs, followed by whole-mount *in situ* hybridization with a *smp30* probe, confirmed the functional importance of these conserved cysteine residues, as well as their antioxidant effects during pronephrogenesis (Fig. 2C,D). These results indicate that the conserved cysteine residues are crucial for pronephros development during *X. laevis* embryogenesis.

RA signaling is critical for pronephrogenesis, and the inhibition of RA signaling results in developmental defects in the pronephros during *X. laevis* embryogenesis^12^. Results of the *in vitro* kidney induction assay demonstrated that Prdx1 functions as a significant modulator of RA signaling during pronephros development. RT-PCR and real time PCR results revealed a decrease in the expression of the pronephric markers *lim1, pax2, smp30*, and *pax8*, which resulted from *prdx1* depletion. The decreased expression of these markers was restored by co-injection with prdx1* RNA (Fig. 3A,B). The functional significance of Prdx1 in RA signaling-mediated pronephrogenesis was further verified by epistatic experiments. Embryos were co-injected with *prdx1* MOs and pronephros-specific RNAs. Whole-mount *in situ* hybridization revealed that the decreased expression of *smp30* was restored by injection with *lim1* RNA, but not with activated *RARα-vp16* or *pax8* (Fig. 3C,D). Taken together, these results show that Prdx1 functions as a downstream mediator of the RA signaling pathway during pronephros development.

In addition, both canonical and non-canonical Wnt signaling pathways are essential for pronephros development in *X. laevis* embryos. The Wnt/β-catenin signaling pathway is crucial for kidney development. An inhibition of this pathway results in abnormalities in the pronephric tubule, glomus, and duct and leads to the reduced expression of pronephros-specific markers^40^. In addition, Wnt/β-catenin independent signaling is responsible for proximal tubule formation during pronephrogenesis^8, 9, 41^.

The role of Prdx1 in Wnt signaling-mediated pronephros development was investigated using Dsh deletion constructs, namely, DshΔDEP and DshΔDIX. Our results showed that the *prdx1* MO-induced inhibition of pronephros formation was rescued by injection with *Dsh* WT mRNA, but not with *DshΔDEP* or *DshΔDIX*. These results indicate that Prdx1 functions upstream of Wnt signaling during pronephrogenesis.

Finally, we explored the mechanism by which Prdx1 regulates Wnt signaling-dependent pronephros development. Cilia are necessary for both canonical and non-canonical Wnt signaling^32–34^. We found that Prdx1 is necessary for primary cilia development in MDCK cells. siRNA transfection and immunofluorescent studies showed that the loss of Prdx1 resulted in a significant reduction in the number of cells with primary cilia (Fig. 4C–E). These data show that Prdx1 plays a critical role in primary cilia formation in MDCK cells and suggest that Prdx1 regulates cilia formation. In addition, we demonstrated that ROS overproduction due to H_2_O_2_ treatment inhibited proximal tubule formation in *X. laevis* embryos. This abnormal phenotype was rescued by *prdx1* overexpression (Fig. 4F,G). These results are consistent with previous findings showing that ROS regulation by Prdx1 is crucial for pronephros development.

In conclusion, our results indicate that Prdx1 is indispensable for pronephrogenesis, which is regulated by RA and Wnt signaling pathways. During pronephros development, Prdx1 modultes RA signaling and Wnt signaling. Our data support the idea that Prdx1 primarily functions in the elimination of excess ROS and it is required for pronephros development, where it presumably acts by modulating ROS levels.

## Materials and Methods

### Ethics statement

This study was performed in strict accordance with the documented standards of the Animal Care and Use Committee and in agreement with international laws and policies (National Institutes of Health Guide for the Care and Use of Laboratory Animals, publication no. 85-23, 1985). The Institutional Review Board of the Ulsan National Institute of Science and Technology approved the experimental use of amphibians (approval no. UNISTACUC-16-14). All members of the research group attended educational and training courses on the proper care and use of experimental animals. Adult aquatic frogs (*X. laevis*), obtained from the Korean *Xenopus* Resource Center for Research, were housed at 18 °C under conditions of 12 h light/12 h dark in approved containers obtained from the Institutional Review Board of the Ulsan National Institute of Science and Technology. There were no unexpected animal deaths during this study.

### Plasmids, MOs, and mRNAs used for the microinjection of *X. laevis* embryos

A cDNA clone encoding full-length *prdx1* was obtained from ATCC (GenBank ID: NM_001092016.1). The *prdx1* MO sequence (25 bp) was 5′-ATG TCT GCC GGA AAC GCA AAA ATT G-3′ (Gene Tools, Philomath, OR, USA). Flag-tagged *prdx1* (wild-type; active-site mutants; and M1, M2, and M1/M2) and HA-tagged *prdx1* were amplified by PCR and subcloned into pCS2 + or pCS107, respectively. For microinjections, *prdx1*, active *rar*, *pax8*, and *lim1* cDNAs were linearized with ApaI, NotI, NotI, and BamHI, respectively. The capped mRNAs were synthesized using the mMessage mMachine Kit (Ambion, Austin, TX, USA) according to the manufacturer’s protocol. mRNAs and the MOs were microinjected into both blastomeres of two-cell stage embryos that were incubated until the desired developmental stage was reached. For the rescue experiments, the MO-resistant mRNAs containing four point mutations in the wobble codons after the ATG start codon were subcloned into pCS107 and then microinjected.

### Whole-mount *in situ* hybridization of *X. laevis* embryos

Embryos at the 33-cell stage were fixed in MEMFA buffer (4% paraformaldehyde in 0.1 M MOPS, 1 mM MgSO_4_, and 2 mM EGTA, pH 7.4) for 2 h at room temperature. To prepare the antisense digoxigenin (Dig)-labeled probe, *prdx1, smp30, xPteg*, and *pax2* cDNAs were linearized with BamHI, NcoI, SacI, and EcoRI, respectively. The probes were generated using the mMessage mMachine Kit and detected with an alkaline phosphatase-labeled anti-Dig antibody (1:1000, Roche, Basel, Switzerland) and BM purple dye.

### RT-PCR and real time PCR

Total RNA was extracted from injected embryos at cell stages ranging from 0 to 40. cDNAs were synthesized by reverse transcription using the PrimeScript First Strand cDNA Synthesis Kit (Takara, Shiga, Japan) according to the manufacturer’s protocol. PCR was carried out using specific primer pairs (Table 1). The PCR products were electrophoresed on 1% agarose gels, and images were then captured using Wise Capture I-1000 software (Daihan Scientific, Seoul, South Korea).

**Table 1:** Primers used for RT-PCR.

Gene	Forward primer (5′ → 3′)	Reverse primer (5′ → 3′)
***odc*** ^44^	**GTCAATGATGGAGTGTATGGATC**	**TCCATTCCGCTCTCCTGAGCAC**
***lim1*** ^45^	**AAGACTCTGAAAGTGCCAATG**	**AGTCTGAGCTTGAGACGATG**
***pax2*** ^27^	**ATGGATATGCACTGCAAGGC**	**TCTTGCTCACACATCCATGG**
***smp30*** ^25^	**ATGGAGGAAGAGTGATCCGCATAGATCCTG**	**TCCGATGTTGCCTGAACACTGAGAGC**
***pax8*** ^45^	**TCAGCTCAGGATGCTCAGC**	**GCTGTAGTAATAGGGATAGC**

For real-time PCR, cDNA templates were amplified with specific PCR primer sets (Table 2) using SYBR Premix Ex Taq according to the manufacturer’s instructions (Takara, Shiga, Japan) and analyzed with a StepOnePlus™Real-Time PCR system (Applied Biosystems, Carlsbad, CA, USA). Relative expression levels of the target genes were analyzed using the comparative Ct (2-DDCt) method^42^. All data are representative of at least three experiments. The Primer3 program was used to design primers and *odc* was used as an internal control^43^.

**Table 2:** Primers used for real time PCR.

Gene	Forward primer (5′ → 3′)	Reverse primer (5′ → 3′)
***odc***	**TGGCTGCACTGATCCACAG**	**TAAAGCCAAGCTCAGCCCC**
***lim1***	**GCCCTGGCAGCAACTATGA**	**CCATTGCACCAAGGGGAGT**
***pax2***	**ATCCGGGACAGGCTTTTGG**	**TGGGGTTGGATGGAATGGC**
***smp30***	**TGGAGGCCCGGATTACTCA**	**CCTCCAGATTGAGGCTGGC**
***pax8***	**CTCTCAAGGCAGTGTGGGG**	**GGAAGTGACACTCCAGGGC**

### Western blot analysis

For western blotting, injected whole embryos were lysed in lysis buffer (137 mM NaCl, 20 mM Tris-HCl, 1% Nonidet-P40, and 10% glycerol, pH 8.0) containing 1 mM phenylmethylsulfonyl fluoride, 5 mM sodium orthovandate, and 1× protease inhibitor cocktail. The lysates were heated for 5 min at 95 °C in loading buffer and then electrophoresed on 12% SDS-PAGE gels. Proteins were cross-reacted with anti-Flag-horseradish peroxidase (HRP) (Sigma, St. Louis, MO, USA) and anti-HA-HRP conjugated (Roche, Indianapolis, IN, USA) antibodies. Immunoreactive bands were detected using the HyGLO™ Kit (Denville Scientific, South Plainfield, NJ, USA).

### Co-immunoprecipitation assay

Lysates from injected embryos were incubated with 2 μg of HA IgG (Santa Cruz Biotechnology, Santa Cruz, CA, USA) in the same buffer used for western blotting for 1 h at 4 °C. The immunocomplexes were pulled down with 15 μL of protein A/G PLUS agarose (Santa Cruz Biotechnology) overnight at 4 °C. Agarose beads were washed thrice in a low-salt lysis buffer. Western blot analysis was performed using anti-Flag-HRP and anti-HA-HRP conjugated primary antibodies.

### MDCK cell culture

MDCK cells were cultured at 37 °C in a humidified atmosphere of 5% CO_2_ in minimum essential medium (MEM) (Gibco, Grand Island, NY, USA) containing 5% fetal bovine serum (Welgene, Seoul, Korea) and 100 U/mL streptomycin/penicillin.

### Immunofluorescence and confocal microscopy

MDCK cells were fixed in 100% methanol in Lab-TEK chambers. Immunofluorescent staining was performed using monoclonal anti-acetylated-tubulin (1:500, Sigma) and Flag (1:500, Applied Biological Materials, Richmond, Canada) antibodies. Alexa Fluor 568 anti-mouse IgG (1:1000, Invitrogen) was used as the secondary antibody. Nuclei were stained with DAPI (4′,6′-diamidino-2-phenylindole, dihydrochloride, 1:5000, Invitrogen), and cells were examined under a Zeiss LSM7 PASCAL confocal microscope. Image processing and analysis were performed with ImageJ and Adobe Photoshop software, respectively.

### Immunohistochemistry

Embryos were fixed in MEMFA buffer for 2 hours at room temperature and then washed with PBS. Immunostaining is performed by using 3G8/4A6 (antibodies specific for proximal tubules/ intermediate, distal and connecting tubules) primary antibodies. Then the embryos were treated with alkaline phosphatase-conjugated anti-mouse IgG + IgM secondary antibodies (Invitrogen).

### Statistical analysis

Data from whole-mount *in situ* hybridization experiments were analyzed using ImageJ software (National Institutes of Health; http://imagej.nih.gov). Results are presented as the means ± standard error (n = 5 with replicates per sample). To determine statistical significance, results were analyzed by one-way ANOVA or unpaired *t*-tests. A p-value of <0.05 was considered statistically significant and is indicated on graphs by an asterisk. p-values of <0.01 and <0.001 are indicated by two and three asterisks, respectively.

## Electronic supplementary material

Supplementary Information
