# Supplementary material for: Peroxiredoxin1, a novel regulator of pronephros development, influences retinoic acid and Wnt signaling by controlling ROS levels
Source: Sci Rep. 2017 Aug 21;7:8874. doi: 10.1038/s41598-017-09262-6 (PMC5567039; doi:10.1038/s41598-017-09262-6)
Supplement: Supplementary file 1 — Supplementary Information [file 41598_2017_9262_MOESM1_ESM.pdf]

Peroxiredoxin1, a novel regulator of pronephros development, influences retinoic acid and Wnt signaling by controlling ROS levels

Soomin Chae[1†], Hyun-Kyung Lee[1†], Yoo-Kyung Kim[1†], Hyo Jung Sim[2], Yoorim Ji[1], Chowon Kim[1], Tayaba Ismail[1], Jeen-Woo Park[1], Oh-Shin Kwon[1], Beom-Sik Kang[1], Dong-Seok Lee[1], Jong-Sup Bae[3], Sang-Hyun Kim[4], Kyoung-Jin Min[5], Taeg Kyu Kwon[5], Mae-Ja Park[6], Jin-Kwan Han[7], Taejoon Kwon[2], Tae-Joo Park[2*], and Hyun-Shik Lee[1*]

[1]KNU-Center for Nonlinear Dynamics, School of Life Sciences, BK21 Plus KNU Creative BioResearch Group, College of Natural Sciences, Kyungpook National University, Daegu 41566, South Korea

[2]School of Life Sciences, Ulsan National Institute of Science and Technology (UNIST), Ulsan 44919, South Korea

[3]College of Pharmacy, Research Institute of Pharmaceutical Sciences, Kyungpook National University, Daegu 41566, South Korea

[4]Department of Pharmacology, College of Medicine, Kyungpook National University, Daegu 41944, South Korea

[5]Department of Immunology, School of Medicine, Keimyung University, Daegu 42601, South Korea

[6]Department of Anatomy, College of Medicine, Kyungpook National University, Daegu 41944, South Korea

[7]Department of Life Sciences, Pohang University of Science and Technology, Pohang, Kyungbuk 37673, South Korea

[†]These authors contributed equally to this work.

[*]Address correspondence to:

H-S Lee, Ph.D. Tel: 82-53-950-7367, Fax: 82-53-943-2762, E-mail: leeh@knu.ac.kr

T-J Park, Ph.D. Tel: 82-52-217-2582, Fax: 82-52-217-5309, E-mail: parktj@unist.ac.kr

Running Title: Peroxiredoxin1 regulates pronephros development

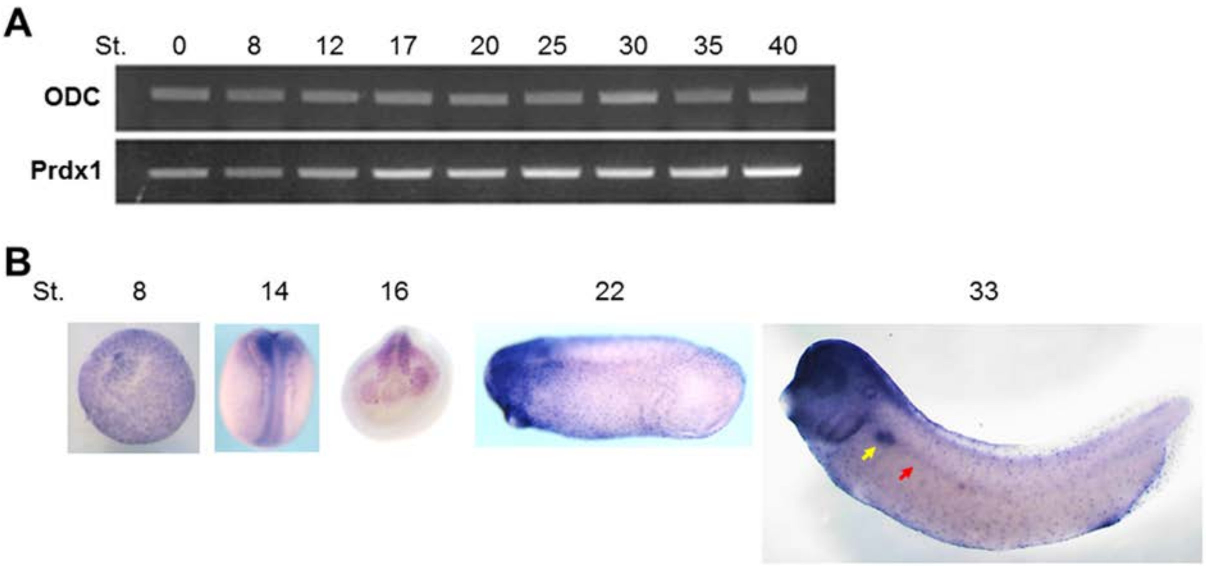

Figure S1. Spatiotemporal expression pattern of Prdx1 during embryogenesis.

A. *Xenopus* embryos were harvested at various stages and RT-PCR was performed using standard methods. The Numbers indicate the embryonic stages. *odc* was used as the loading control. The expression of *prdx1*, a maternal gene, gradually increased from the blastula to the tadpole stage.

B. Whole mount *in situ* hybridization with a digoxigenin labeled antisense probe against *prdx1* was performed for embryos at stage 8, 14, 16, 22 and 33. *prdx1* was expressed in the forebrain, eye, multiciliated cells, and pronephros. The yellow arrow points to the developing pronephros; the red arrow points to the presumptive pronephric tubule ducts.

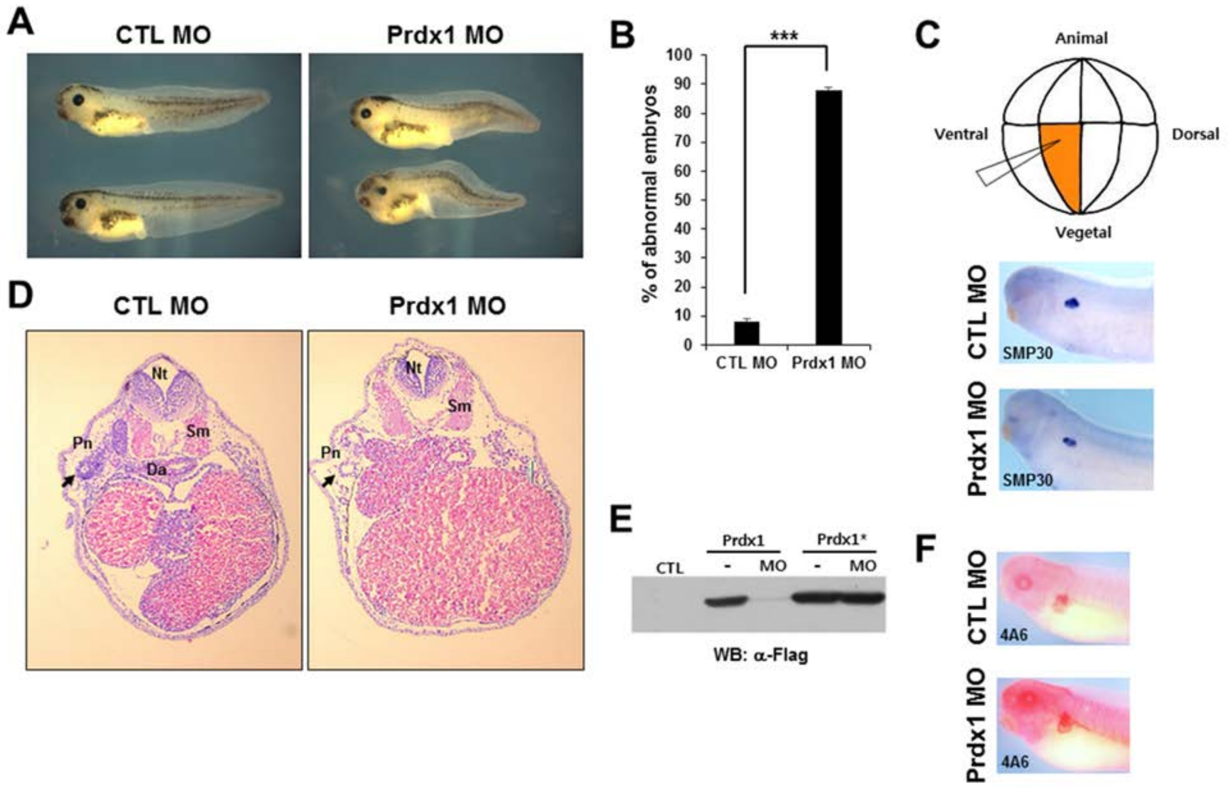

Figure S2. Injection of the *prdx1* MOs results in phenotypic abnormalities.

A. *prdx1* MOs were injected into both blastomeres of two-cell stage embryos. There were phenotypic abnormalities in *prdx1* MO-injected embryos compared with control MO-injected embryos.

B. Severity of the phenotypic abnormalities in *prdx1* MO-injected embryos compared with control MO-injected embryos.

C. *prdx1* MO was injected into a V.2.2 blastomere of 16-cell stage embryos. Embryos at the stage 33 were used for whole-mount *in situ* hybridization, and proximal tubules in the pronephros were visualized with a *smp30* probe. Embryos exhibited similar developmental abnormalities as were observed for *prdx1* MO injections at two-cell stage.

D. *prdx1* MOs (40 ng) were injected into both blastomeres of two-cell stage embryos. Embryos at the stage 33 were transversely sectioned, and serial sections were stained with hematoxylin and eosin. The *prdx1* MO-injected embryos displayed malformed or undifferentiated internal organs that appeared to be scattered. Nt, neural tube; Sm, somites; Pn, pronephros; Da, dorsal aorta.

E. Specificity of *prdx1* MO was confirmed by western blot analysis using Flag antibody. The translation product for WT *prdx1* RNA was markedly reduced by *prdx1* MO.

F. 4A6 staining of intermediate, distal and connecting tubules was performed at stage 40 in *prdx1* morphants. *prdx1* knockdown did not affected formation of intermediate, distal and connecting tubules.

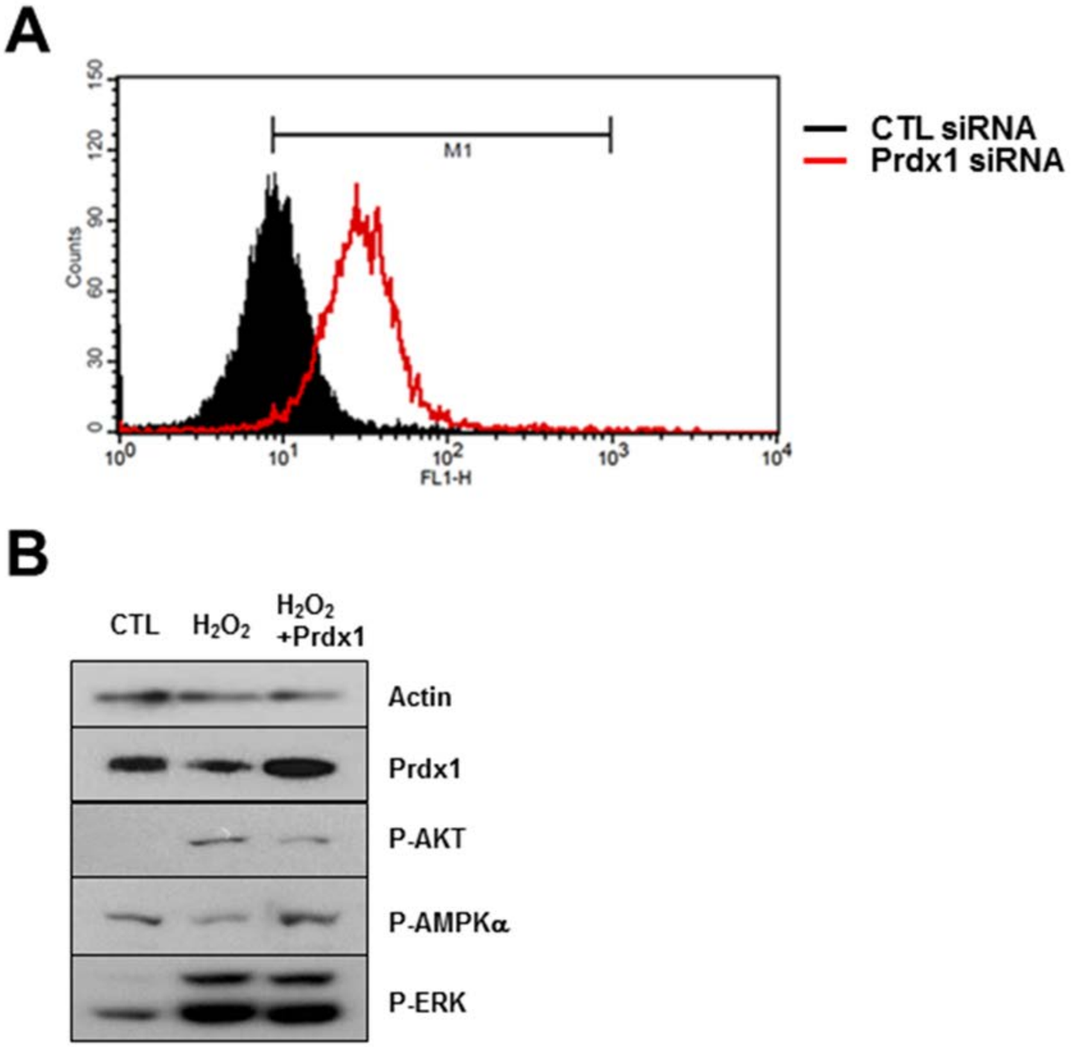

Figure S3. Transfection with Prdx1 siRNAs increased the ROS levels in MDCK cells.

A. Endogenous ROS levels in MDCK cells transfected with either 10 nM *prdx1* or control siRNAs were measured using flow cytometry. The cellular ROS level was higher in *prdx1* siRNA-transfected cells than in control siRNA-transfected cells.

B. MDCK cells were co-treated with H_2O_2 and H_2O_2 + *prdx1* and observed their effects on ROS products. Treatment of H_2O_2 enhanced the phosphorylation of AKT (P-AKT) while phosphorylation of AMPKα (P-AMPKα) was reduced. Prdx1 transfection reversed the expression *i.e.* downregulated the expression of P-AKT and upregulated the P-AMPKα

expression. Induced P-ERK by H_2O_2 was not affected by expression of *prdx1* in transfected MDCK cells.

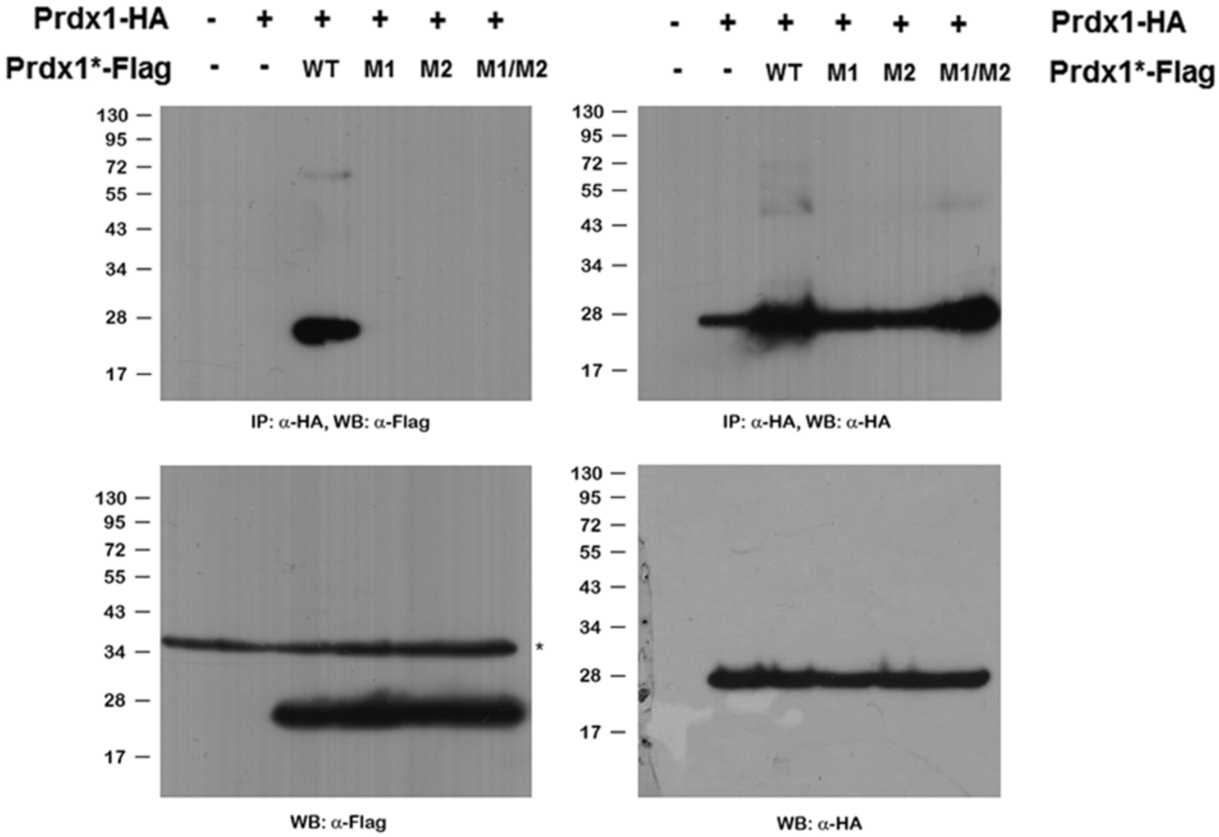

Figure S4. Selected representative full gel scans.

Full gel scans of figures 2B. Asterisk indicates non-specific band.
